# Synthesis and Modification of Gelatin Methacryloyl (GelMA) with Antibacterial Quaternary Groups and Its Potential for Periodontal Applications

**DOI:** 10.3390/gels8100630

**Published:** 2022-10-05

**Authors:** Nelson Vargas-Alfredo, Marta Munar-Bestard, Joana Maria Ramis, Marta Monjo

**Affiliations:** 1 Cell Therapy and Tissue Engineering Group, Department of Fundamental Biology and Health Sciences, Research Institute on Health Sciences (IUNICS), University of the Balearic Islands, Ctra. Valldemossa Km 7.5, 07122 Palma de Mallorca, Spain; 2 Health Research Institute of the Balearic Islands (IdISBa), Ctra. Valldemossa 79, University Hospital Son Espases, Edificio S, 07120 Palma de Mallorca, Spain

**Keywords:** gelatin methacryloyl hydrogels, quaternary groups, antibacterial hydrogels, periodontal hydrogels, regenerative hydrogels

## Abstract

Gelatin methacryloyl (GelMA) hydrogels have been widely used for different biomedical applications due to their tunable physical characteristics and appropriate biological properties. In addition, GelMA could be modified with the addition of functional groups providing inherent antibacterial capabilities. Here, GelMA-based hydrogels were developed through the combination of a GelMA unmodified and modified polymer with quaternary ammonium groups (GelMAQ). The GelMAQ was synthesized from GelMA with a low degree of substitution of methacrylamide groups (DSMA) and grafted with glycidyltrimethylammonium chloride in the free amine groups of the lysine moieties present in the original gelatin. GelMAs with high DSMA and GelMAQ were combined 50/50% or 25/75% (*w*/*w*), respectively, and compared to controls GelMA and GelMA with added chlorhexidine (CHX) at 0.2%. The different hydrogels were characterized using ^1^H-NMR spectroscopy and swelling behavior and tested in (1) *Porphyromonas gingivalis* to evaluate their antibacterial properties and (2) human gingival fibroblast to evaluate their cell biocompatibility and regenerative properties. GelMA/GelMAQ 25/75% showed good antibacterial properties but also excellent biocompatibility and regenerative properties toward human fibroblasts in the wound healing assay. Taken together, these results suggest that the modification of GelMA with quaternary groups could facilitate periodontal tissue regeneration, with good biocompatibility and added antibacterial properties.

## 1. Introduction

The periodontal disease includes various degenerative and inflammatory processes of the gums (gingivitis), periodontal ligaments, alveolar bone, and dental cementum [1,2,3,4]. It is one of the most prevalent chronic diseases in the world [5]. Therefore, it meets the criteria to be considered a public health problem: (1) it is widespread affecting more than 50% of the adult population, and the world health organization reported that between 10–15% of the world population suffers from severe periodontitis, making this disease the sixth most prevalent in humanity; (2) periodontitis seriously affects people′s quality of life, their self-esteem, and their general well-being. Moreover, periodontal disease is known to increase with age and the incidence increases rapidly in adults aged 30 to 40 years [3,6,7,8,9,10,11]. The main cause is the bacterial growth that occurs due to the accumulation of subgingival plaque [1,3]. These bacteria release byproducts and enzymes such as collagenase, fibrinolysin, and others that destroy the periodontium [12,13]. Oral hygiene brushing and flossing are of great importance to prevent this condition since when plaque adheres to teeth, tartar cannot be removed by daily brushing [11]. Later, the bacteria begin to invade deep tissues [3], and the collagen and ligaments that hold teeth firm are degraded. This creates a space between the gum and the tooth, where the periodontal cavity is formed, thus causing periodontitis [3]. In addition to hygiene habits, it is known that there are other risk factors for periodontal disease such as smoking, alcohol, malnutrition, diabetes, osteoporosis, stress, lack of exercise, obesity, cardiovascular diseases, and hormonal variations due to pregnancy and menopause [12,14,15,16,17,18,19].

Various surgical and non-surgical treatments can be used depending on the severity of the disease. Scaling and root planning are the gold standard of non-surgical treatments [20]. However, these procedures have some shortcomings, such as the inability to reach the deeper pockets and furcations. Therefore, to completely cure periodontitis, antibiotics must be given in conjunction with these procedures. Therapeutic success or failure is determined, in addition to the antimicrobial action of the drug, by the site of infection, the transporter system, and the form of drug release [21]. Oral administration of antibiotics has many systemic side effects: the concentration of drugs reaching the periodontal pocket is inadequate, and it drops rapidly to a subtherapeutic level, requiring frequent dosing; in addition, microbial resistance develops [22]. Because the disease is confined to the periodontal cavity, local release of the drug into the pocket itself is the best option. Some of the advantages of local drug administration are: direct action on the site of the disease; better therapeutic efficacy of the drug; prolonged effect of the action; non-invasive, painless, and simple application [3].

Using a variety of polymers, different formulations have been made to provide high stability, non-toxicity, biocompatibility, non-immunogenicity, and biodegradability. The most used products for the controlled release of drugs in periodontitis are gels (hyaluronic acid, polycaprolactone (PCL), etc.), fibers (alginate, ethylene vinyl acetate, etc.), strips (PCL, ethylcellulose, etc.), films (chitosan(CH)/PCL, poly(DL-lactide-co-glycolide(PLGA)), etc.) and microparticles/microspheres (PCL, CH, PLGA), that include different drugs such as simvastatin, metronidazole, chlorhexidine (CHX), ciprofloxacin, tetracycline, gentamicin, moxifloxacin, amoxicillin, doxycycline, minocycline, and silver [1,3,20,23,24].

Of all the dosage systems used, gels are the ones that offer the most advantages. The gels are versatile materials and easy to prepare and administer. They are also more biocompatible and bioadhesive, which helps their adherence to the mucosa in the dental pocket. Hydrogels are hydrophilic polymeric networks that structurally consist of 3D cross-linked chains. These materials can absorb 10–20 times their weight in water [25]. Due to these characteristics, hydrogels are materials that function efficiently as bioadhesive reservoirs for drugs and bioactive substances [26,27].

In recent years, research has been carried out on the use of natural gels that can be used to release substances with a positive impact on cell proliferation. For example, gelatin has been used to make hydrogels for the controlled release of bone morphogenetic protein-2 (BMP-2) and also growth factors from platelet-rich plasma (PRP) for the regeneration of periodontal tissue [28,29,30]. Gelatin is a natural polymer derived from collagen, the major component of ECM in most tissues, with a variety of bioactive motifs, such as arginine-glycineaspartic acid (RGD) [31], which promotes cell attachment, and its matrix metalloproteinase (MMP) sensitive moieties, which are suitable for cell remodeling [31,32]. Likewise, methacrylated gelatin (GelMA) hydrogels have also been used, for instance, for the release of encapsulated human periodontal ligament stem cells for the regeneration of alveolar bone defects [33], making it a potentially attractive material for tissue engineering applications, as it has been reported to promote cell proliferation and migration. [31,34] GelMA is a material that not only has biocompatibility and biodegradability but is also inexpensive, non-cytotoxic, and non-immunogenic. These properties make it an ideal biomaterial for the manufacture of biological scaffolds and controlled release systems [33].

In this study, we investigated the modification of GelMA with the addition of quaternary functional groups (GelMAQ) with the aim of providing antibacterial properties while maintaining its biocompatibility. After synthesizing and characterizing the different polymers with ^1^H-NMR spectroscopy and swelling behavior, different hydrogels were produced and tested. GelMA with a high degree of substitution of methacrylamide groups (DSMA) and GelMAQ were combined 50/50% or 25/75% (*w*/*w*), respectively, and compared to controls GelMA and GelMA with added CHX at 0.2%. The biocompatibility and regenerative properties of the synthesized hydrogels were tested on human gingival fibroblasts under inflammatory conditions. *Porphyromonas gingivalis* growth, viability and gingipain activity were also studied as antibacterial effects of hydrogels.

## 2. Results and Discussion

### 2.1. Gelatin Methacryloyl (GelMA) and Gelatin Methacryloyl Quaternary (GelMAQ) Synthesis

**Figure 1:**
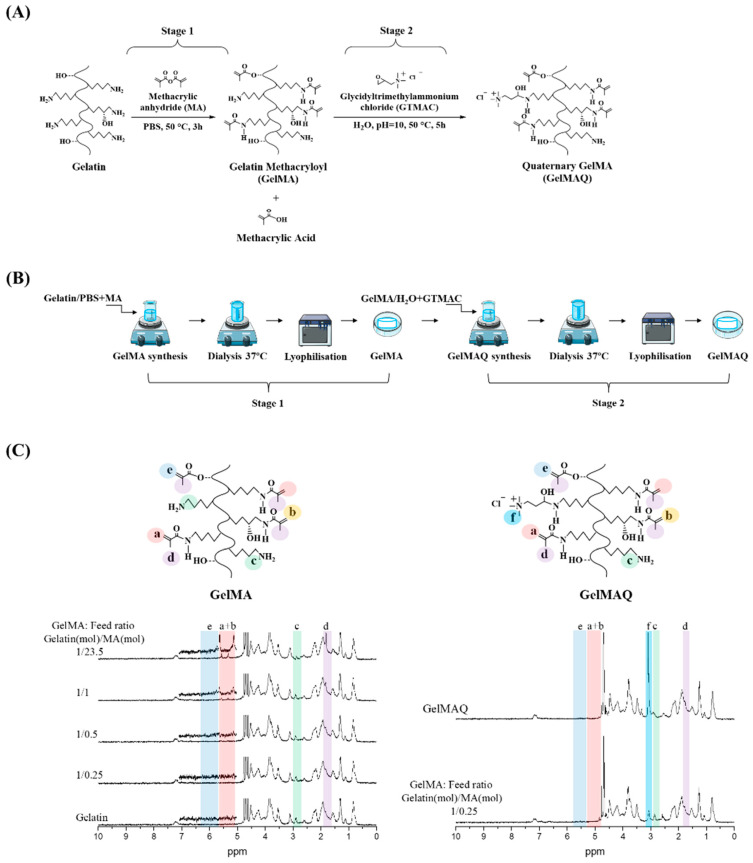
Gelatin Methacryloyl (GelMA) and Gelatin Methacryloyl Quaternary (GelMAQ) synthesis and characterization. (**A**) Stage 1: GelMA synthesis by methacrylation of porcine gelatin with MA at 50 °C in PBS. Stage 2: GelMAQ synthesis by glycidyltrimethylammonium chloride (GTMAC) reaction with GelMA at 50 °C and pH = 10. (**B**) General experimental procedure for GelMA and GelMAQ synthesis. (**C**) Stage 1: ^1^H NMR characterization of gelatin and GelMAs synthesized with different gelatin/MA molar ratios to confirm the substitution of primary amine groups by methacryloyl groups. The assignation of signals was as follows: signal at δ = 1.8 ppm assigned to methyl groups of MA (colored in purple, letter d), the signal at δ = 2.9 ppm assigned to methylene protons of lysine (colored in green, letter c), the signal between δ = 5.3–5.5 ppm assigned to vinylic protons of MA (colored in pink, letters a and b), the signal at δ = ppm assigned to another vinylic protons to MA (colored in blue, letter e) Stage 2: 1H NMR GelMA and GelMAQ characterization to confirm the substitution of primary amine groups by quaternary ammonium groups from the GTMAC. In this case, the signal at δ = 3.1 ppm was assigned to quaternary groups (colored in cyan, letter f). Chemical structures from ChemDraw software, procedures figures from smart servier medical art and graph spectrum processed with Origin software.

GelMAQ is a gelatin derivative synthesized from GelMA and modified with quaternary ammonium groups (-NR3+). GelMAQ synthesis was carried out in two stages as it is shown in Figure 1A, adjusting the protocols reported in the literature [35,36]. In stage 1, GelMA was synthesized by the typical reaction between methacrylic anhydride (MA) and porcine gelatin. In stage 2, GelMAQ was synthesized by the reaction between glycidyltrimethylammonium chloride (GTMAC) and GelMA. The laboratory procedure also is shown in Figure 1B. 

During the synthesis of GelMA, the incorporation of MA into gelatin is carried out at the free amino functional groups (-NH2) of the lysine residues of the gelatin [31]. Precisely these are the acrylate groups that, in the presence of an initiator, polymerize to give the corresponding hydrogel. Therefore, the higher the degree of substitution (DS) by MA, the greater the degree of crosslinking, and the more rigid hydrogels are obtained. However, in these conditions, there are practically no free amino groups left for subsequent reactions. On the other hand, GelMA with a lower DS by MA has a large number of free amino functional groups, and softer hydrogels with a poor degree of crosslinking are obtained.

Therefore, in our approach, it was very important to have a large number of free amino groups for subsequent modification of GelMA, without losing the crosslinking capability. To the best of our knowledge, this is the first study reporting GelMA modification with quaternary ammonium groups. Consequently, to find out the most adequate DS of GelMA, we carried out in stage 1 the synthesis of several GelMAs with a different molar ratio of gelatin/MA.

Figure 1C shows the ^1^H NMR characterization of the synthesized GelMAs. First, all the signals in the spectra correspond to the GelMA structure according to the literature [37,38]. In general, there are four regions, of which three are the most evident. The signals at δ = 5.3–5.5 ppm (colored in pink) correspond to the vinylic protons from the MA groups; the signals at δ = 2.9 ppm (colored in green) correspond to methylene protons from the lysine groups in the gelatin, and the signals at δ = 1.8 ppm (colored in purple) correspond to methyl groups of the MA groups. Second, in comparison, the 1H NMR spectra clearly showed the trend of increasing DS depending on the molar ratio gelatin/MA. Thus, the presence of MA groups gradually increased the signal at δ = 5.3–5.5 ppm while gradually decreasing the signal at δ = 2.9 ppm. Therefore, GelMA with different DS were obtained according to 1H NMR (Table 1), which agrees with the amounts of MA used for the synthesis and the reaction conditions. There are reports in the literature in which with similar amounts of MA, a faster reaction with higher DS is reached under alkalinity conditions (pH = 9) [37]. In this case, in contrast to the literature, we decided to use reaction conditions at neutral pH to have longer times and thus to be able to better control the DS at constant reaction times. Carrying out the reactions at a constant time of 3 h, we obtained GelMA with a DS of about 6% minimum and about 76% maximum (Table 1). The minimum DS was obtained when 25% mols of the MA (1.18 mM) were used with respect to the total amount of lysine-mol equivalents present in the gelatin [37]. The maximum degree was obtained when a 23-fold molar excess of MA (111.88 mM) with respect to the amount of lysine equivalents was used. The swelling (Sw (%)) of the hydrogels showed that the water absorption is approximately similar due to the high hydrophilicity of the gelatin and GelMA (Table 1).

From the obtained results for the GelMA synthesis in stage 1, we chose the GelMA with DS = 6% obtained with a feed molar ratio gelatin/MA = 1/0.25 (Table 2) for the GelMAQ synthesis in stage 2. This GelMA is the one with the highest amount of free amino groups that in the presence of I2959 under UV irradiation polymerized in a relatively short time (10 min). The GelMAQ synthesis was carried out in soft conditions but at an alkaline pH = 10 to activate the ring opening of the epoxide group in the GTMAC [36]. The GelMAQ was characterized by ^1^H NMR, and the comparison spectra before (GelMA) and after reaction (GelMAQ) are shown in Figure 1C. According to ^1^H NMR, GelMAQ was successfully obtained. The signal in the region between δ = 2.5–3.5 ppm confirms this modification. This signal corresponds to protons from quaternary groups. The signal at δ = 2.9 ppm (colored in green) corresponds to methylene groups from free amino groups in the original gelatin. These groups react with the epoxide group of GTMAC and diminish the intensity. In the 1H NMR spectra of GelMAQ, it is observed through the appearance of a new signal at δ = 3.1 ppm (colored in cyan, letter f) and the decrease in the signal at δ = 2.9 ppm (colored in green, letter c). The percentage of incorporation of GTMAC is not easy to determine because the ^1^H NMR signal in GelMAQ is overlapped with the initial GelMA. Additionally, the Van Slyke method typically used to determine free amino groups in GelMA is not feasible due to the presence of quaternary groups [39].

### 2.2. Hydrogel Synthesis

**Figure 2:**
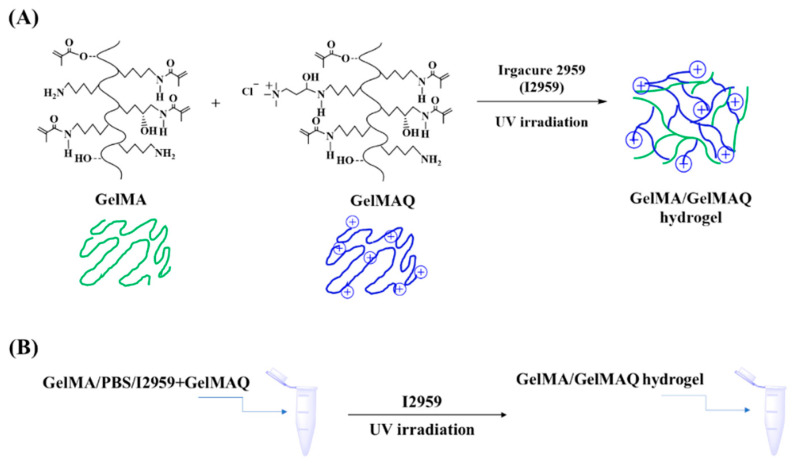
GelMA/GelMAQ hydrogel synthesis. (**A**) Synthetic chemical route for the GelMA/GelMAQ hydrogel synthesis, using I2959 as a crosslinker and under UV irradiation. (**B**) General experimental procedure to GelMA/GelMAQ hydrogel synthesis.

GelMAQ was not miscible with the I2959 crosslinker due to its high hydrophilicity. Alternatively, we used mixtures of higher DS non-quaternary GelMA and GelMAQ for its formulation [40]. Through this method it was possible to obtain the hydrogels. GelMAQ hydrogel synthesis was carried out as shown in Figure 2.

These mixes were made with variable content of both polymers in order to have on one hand samples with more quaternary groups and, on the other hand, to observe the effect of minimum GelMA required to polymerize GelMAQ. We synthesized two series of hydrogels, both mixing the GelMAQ with non-quaternary GelMA. We used the GelMA synthesized with a molar ratio gelatin/MA = 1/23.5 as non-quaternary GelMA in the mixes. This GelMA was used because it polymerizes easily due to its higher DS = 76% (Table 1). We observed that the presence of quaternary groups in GelMAQ affected the polymerization. The presence of GelMAQ decreased the I2959 solubility, and the cationic groups in its chemical structure increased the electrostatic repulsion of these groups, consequently increasing the time of polymerization. The time of polymerization until obtaining the hydrogel was 2 min for GelMA, 30 min for GelMA/GelMAQ 50/50%, and 90 min for GelMA/GelMAQ 25/75%. The content of GelMAQ increased the polymerization time, and even no polymerization was achieved when using 100% of GelMAQ. In this case, it is important to remark that the increase of the reaction time would not limit the potential commercialization of these gels, either for GelMA/GelMAQ 50/50% or 25/75%, since they can be commercialized after polymerization, avoiding this disadvantage, and making it easier to handle. These gels are meant to be applied in the gum as a low viscosity hydrogel, so these polymers were synthesized with poor mechanical properties. Mechanical/morphological properties and in vitro enzymatic degradation were not considered in the present study, although they have already been reported for GelMA [38].

In this manner, for the following biological studies, we used GelMA and two modifications of GelMA with quaternary groups: GelMA/GelMAQ 50/50% (*w*/*w*) with Sw (%) = 1264.9 and GelMA/GelMAQ 25/75% (*w*/*w*) with Sw (%) = 1498.2.

### 2.3. In Vitro Biological Properties Evaluation

#### 2.3.1. Effect of the Different Formulations of GelMA on Biocompatibility, Wound Closure Assay and Gene Expression in Human Gingival Fibroblasts

In the last few years, a number of studies have tested commercially available oral gels containing CHX or other antimicrobial agents, having raised concerns about their cytotoxicity on periodontal tissues. CHX is the most widely used antiseptic on the market and it has been shown to have adverse effects, such as parotid gland swelling, pigmentation of the oral soft tissues and teeth, type 1 hypersensitivity reactions, taste alteration, burning sensation, oral mucosa ulceration or erosions, a transient anesthetic sensation, and paresthesia, which have limited its acceptance and prolonged use [41,42]. Furthermore, current studies have demonstrated the high toxicity of CHX on human gingival fibroblasts, suggesting that it may cause oral tissue damage if used continuously and at high concentrations [43]. In fact, after prolonged use, a high concentration of p-chloroaniline has been detected in saliva, which is potentially carcinogenic [44]. Therefore, in this article, we explored the possibility of having a hydrogel with intrinsic antibacterial activity and high biocompatibility.

First, we tested the biocompatibility and regenerative effect of GelMA and two modifications of GelMa with quaternary groups: GelMA/GelMAQ 50/50% (*w*/*w*) and GelMA/GelMAQ 25/75% (*w*/*w*). As a positive control, we used GelMA with added CHX at 0.2%. These gels were tested on human gingival fibroblasts under inflammatory conditions to simulate periodontitis. To set inflammation, cell cultures were treated with 1 µg/mL of lipopolysaccharide (LPS) from *P. gingivalis.* LPS is a virulence factor, that can elicit important host response reactions, such as stimulation of inflammatory cytokines and chemokines expression, playing a fundamental role in the development of periodontal disease pathogenesis and regulation of tissue homeostasis [45]. Additionally, in the cell cultures, a scratch was performed, and cells were treated with the different GelMA gels at 5% to evaluate cytotoxicity, wound healing, and gene expression.

**Figure 3:**
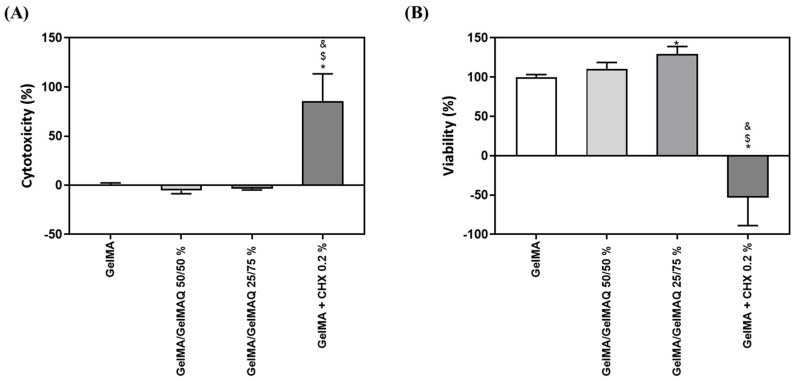
In vitro biocompatibility study of different types of GelMA on human gingival fibroblasts: (**A**) LDH activity, a cytotoxicity indicator, measured in culture media after the application of different types of GelMA for 48 h. Control negative (0% toxicity) was obtained from cells with GelMA. Control positive (100% toxicity) was obtained from cells cultured on plastic and treated with 1% Triton X-100; (**B**) metabolic activity, an indicator of the viability of cells, measured in cultured media after the application of different types of GelMA for 48 h. Control negative (100% viability of cells) was obtained from cells with GelMA. Values represent the mean ± SEM (n = 6). Results were statistically compared by ANOVA and LSD as a post hoc: * *p* < 0.05 treatment vs. GelMA. $ *p* < 0.05 treatment vs. GelMA/GelMAQ 50/50%. & *p* < 0.05 treatment vs. GelMA/GelMAQ 25/75%.

GelMA has been shown in previous reports to be biocompatible, non-cytotoxic, and non-immunogenic, making it an ideal biological scaffold material [33]. In this article we have used two assays to check the toxicity and biocompatibility of the different modifications of GelMA gels: the LDH assay was used as an indicator of cytotoxicity, as this enzyme leaks out through the plasma membrane of damaged cells, and of the metabolic activity of the cells, which is an indicator of cell viability. As shown in Figure 3A,B, GelMA + CHX 0.2% produced significant high levels of cytotoxicity and significant lower metabolic activity compared to the other treatments, which exceeds the maximum value allowed according to the ISO 10,993:5. This is in accordance with a previous study, showing that most commercial periodontal gels containing CHX exhibit high cytotoxicity on gingival fibroblasts [46]. In contrast, GelMA, GelMA/GelMAQ 50/50%, and GelMA/GelMAQ 25/75% showed good biocompatibility on gingival fibroblasts and were not cytotoxic according to ISO 10,993:5. Interestingly, GelMA/GelMAQ 25/75% showed improved results of metabolic activity on gingival fibroblasts compared to GelMA suggesting that this modification improves biocompatibility. In another study, using a GTMAC-modified chitin gel, GTMAC was considered to have low cytotoxicity, and fibroblasts presented a normal shape when in contact with the gel [47].

**Figure 4:**
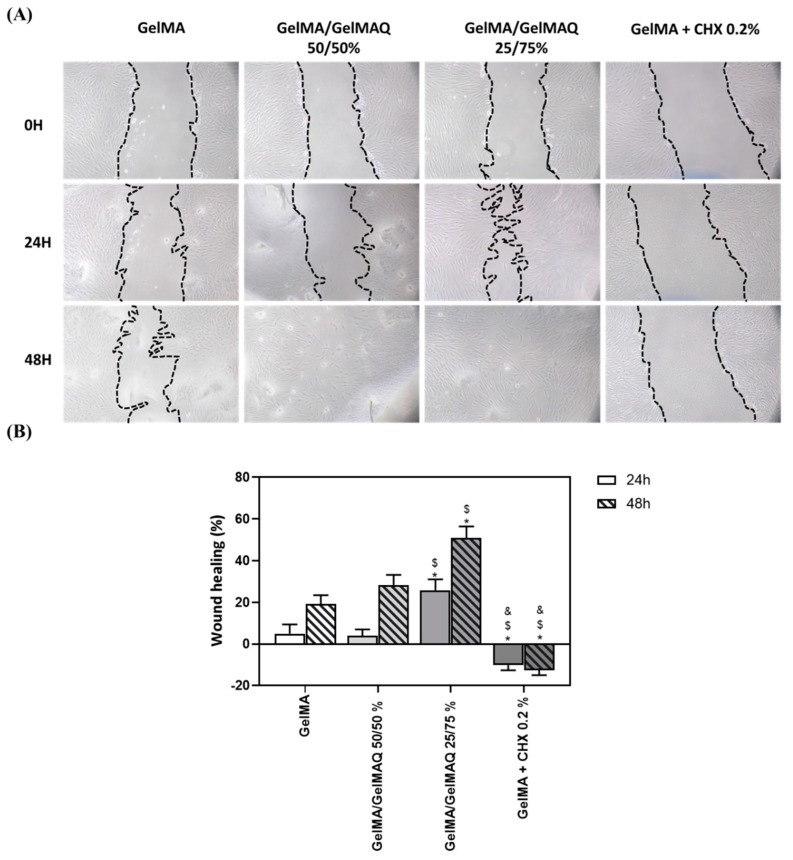
Wound healing assay after treatment with the different types of GelMA on human gingival fibroblasts: (**A**) images of wound healing 24 and 48 h after treatment; a magnification of 100× was used to take the images; (**B**) quantification of the % of wound closure area 24 h and 48 h after treatment. Quantification was performed with ImageJ. Values represent the mean ± SEM (n = 6). Results were statistically compared by ANOVA and LSD as post hoc: * *p* < 0.05 treatment vs. GelMA. $ *p* < 0.05 treatment vs. GelMA/GelMAQ 50/50%. & *p* < 0.05 treatment vs. GelMA/GelMAQ 25/75%.

Different studies have shown that GelMA hydrogels have adjustable physicochemical properties that make them widely applicable to promote high-quality tissue regeneration [48]. Due to the large number of regenerative properties that this biomaterial presents, in this article, we have carried out a wound healing study on human gingival fibroblasts at different times to verify the regenerative potential of the different GelMA gels produced (Figure 4A). The pictures show that 24 h and 48 h after applying the different gels, cells treated with the Gel that contain more GTMAC (GelMA/GelMAQ 25/75%) showed greater wound closure, compared to GelMA and GelMA/GelMAQ 50/50% (Figure 4B). These results might be due to the described effect of GTMAC as an effective wound healing promotor by stimulating the secretion of cytokines and facilitating cell proliferation. [47,49] Therefore, the migration response of gingival fibroblasts requires investigation when evaluating any regenerative agent. However, the factors that affect periodontal tissue regeneration are complex, and the direct equivalence of fibroblast cell culture with in vivo applications is limited. A more complex evaluation of the regenerative capacity of the gel and the use of more complex wound healing models, such as ex vivo gingiva explants or three-dimensional models of the oral mucosa, or in vivo animal models, should be performed. On the other hand, decreased wound healing was observed in cells treated with GelMA + CHX 0.2% compared to the other groups. This observation is consistent with the results observed with the cytotoxic reported effects of CHX and inhibition of fibroblasts proliferation [46]. Under the microscope (Figure 4A), fibroblastic cells treated with GelMA, GelMA/GelMAQ 50/50%, and GelMA/GelMAQ 25/75% presented normal cell shape and spreading. This indicated that the incorporation of GTMAC to GelMA does not alter GelMAs reported biocompatibility [47].

**Figure 5:**
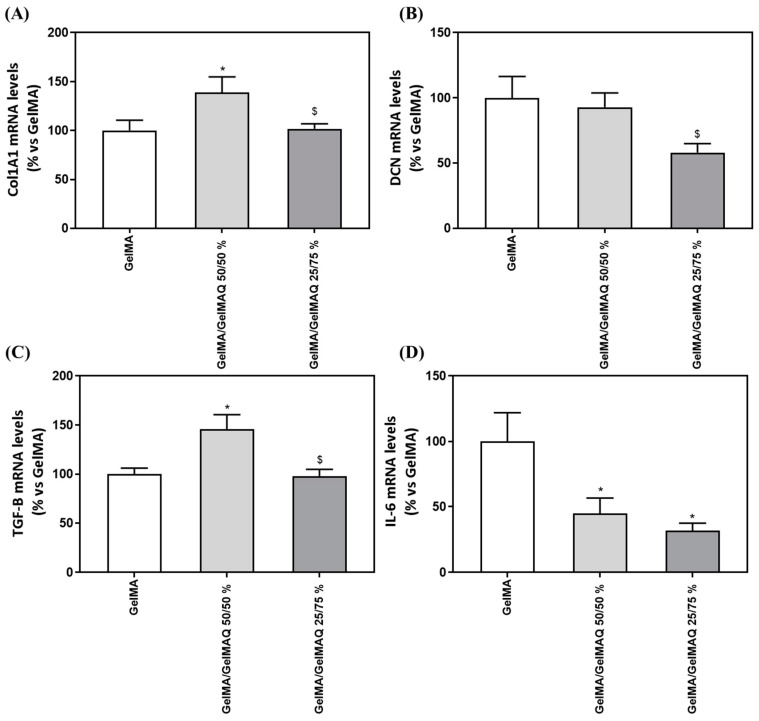
Gene expression levels of marker genes after treatment with GelMA gels. Effect of treatment with GelMA, GelMA/GelMAQ 50/50% and GelMA/GelMAQ 25/75% for 48 h after wound healing on mRNA expression levels of COL1A1 (**A**), DCN (**B**), TGF-B (**C**), and IL-6 (**D**) in iHGF, in the presence of LPS. Negative control (C−) was obtained from cells seeded on plastic treated with GelMA; only the biocompatible gels were tested compared to the negative control since the GelMA + CHX 0.2% produced cell death and cell monolayer disruption. Results are expressed as % vs. GelMA which was set to 100%. Values represent the mean ± SEM (n = 9). Results were statistically compared by Kruskal–Wallis for DCN and TGF-B1; and by ANOVA and LSD as post hoc for Col1A1 and IL-6: * *p* < 0.05 treatment vs. GelMA. $ *p* < 0.05 treatment vs. GelMA/GelMAQ 50/50%.

We next evaluated the regenerative properties of the three hydrogels that were not cytotoxic to the cells, since the GelMA + CHX 0.2% gel produced cell death and RNA could not be isolated, by analyzing gene expression levels of different markers (Table 3) related to the production of the ECM components (COLA1A1 and DCN), to wound healing/fibrogenic agent (TGF-B1) and inflammation (IL-6). Interestingly, cells treated with GelMA/GelMAQ 50/50% showed a significant increase in Collagen I α1(COL1A1) compared to the GelMA (Figure 5A), which is associated with increased human gingival fibroblast differentiation and decreased scars during wound healing [50]. Thus, this gel could treat periodontal disease, characterized by an increase in collagen degradation, leading to loss of connective tissue [51].

Decorin (DCN), a small cellular proteoglycan highly expressed in human gingiva that regulates the organization of collagen fibrils, including type I and type III [52], showed decreased gene expression after GelMA/GelMAQ 25/75% treatment compared to GelMA/GelMAQ 50/50% (Figure 5B). DCN expression has inversely been associated with cell proliferation in regenerating gingival fibroblasts, which may be related to our findings. Thus, it is up-regulated when cells reach quiescence or are subjected to growth inhibition [53]. These results agree with our finding that lower DCN levels are found in cells treated with GelMA/GelMAQ 25/75% which also showed a significantly increased wound closure.

The role of fibroblasts to organize and produce ECM components in proportional amounts in accordance with healthy tissue is critical for proper wound healing [51]. Cells treated with GelMA/GelMAQ 50/50% showed a significant increase in Transforming growth factor-β1 (TGF-β1) compared to the GelMA (Figure 5C). TGF-β1 is the principal activator of collagen expression and other key components of ECM during the healing process [51], and it has also been shown to stimulate the proliferation of fibroblasts and the synthesis of proteins of extracellular matrix (ECM) [54]. Cytokines play an important role in the pathophysiology of chronic inflammatory diseases, including periodontitis. Interleukin-6 (IL-6) in particular is a major mediator of the host response to tissue injury, infection, and bone resorption. [55] IL-6 expression decreased significantly after treatment with GelMA/GelMAQ 50/50% and GelMA/GelMAQ 25/75% compared to GelMA (Figure 5D), which is interesting as it added an anti-inflammatory effect, besides its regenerative effect. IL-6 is a critical component of the acute-phase response because it triggers the release of C-reactive protein (CRP), which is involved in immune response and pro-inflammatory host reactions [56].

#### 2.3.2. Effect of the Different Formulations of GelMA on Bacterial Growth and Gingipain Activity of *P. gingivalis*

The antimicrobial activity of the different GelMA gels on the bacteria growth rate, viability, and gingipain activity were also evaluated. *P. gingivalis* was used in the present investigation, as it is considered a keystone pathogen in periodontitis, producing a broad range of virulence factors, including gingipains that contribute to periodontal tissue destruction either directly or indirectly by modulating the host inflammatory response [57].

**Figure 6:**
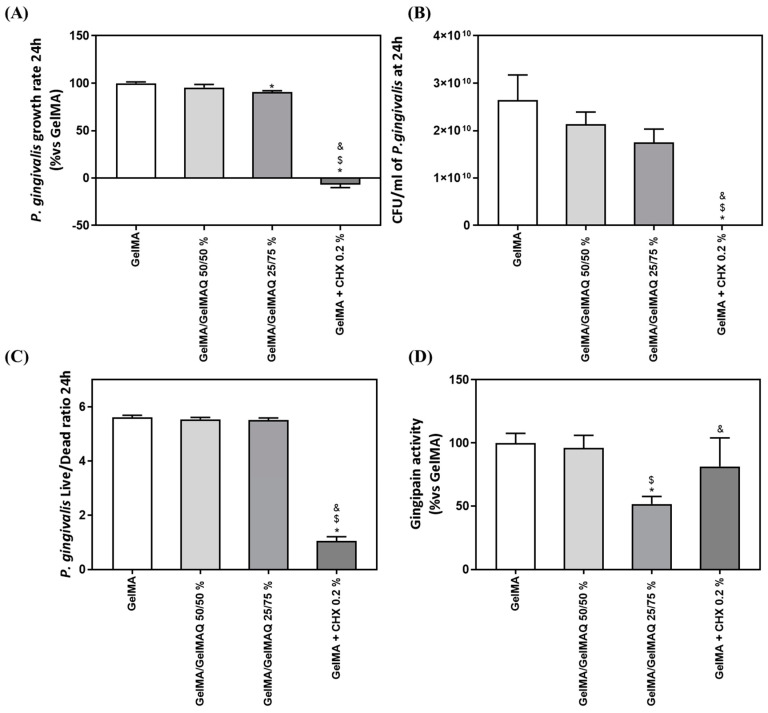
Antimicrobial activity of different GelMA gels: (**A**) *P. gingivalis* growth rate cultured with different GelMA gels (n = 6); (**B**) number of *P. gingivalis* CFU/mL after 24 h of incubation with the different treatments (n = 6); (**C**) *P. gingivalis* Live/Dead Ratio treated for 24 h with different GelMA gels; (**D**) in vitro gingipain activity from *P. gingivalis* after 24 h of treatment (n = 6). Results are expressed as % vs. GelMA which was set to 100%. Data represent the mean ± SEM. Negative control (C−) was bacterial suspension with GelMA treatment and Positive control (C+) was bacterial suspension with GelMA + CHX at 0.2%. Results were statistically compared by ANOVA and LSD as post hoc: * *p* < 0.05 treatment vs. GelMA. $ *p* < 0.05 treatment vs. GelMA/GelMAQ 50/50%. & *p* < 0.05 treatment vs. GelMA/GelMAQ 25/75%.

The exponential growth phase of *P. gingivalis* was established from 0 to 24 h of culture, to estimate the growth rate of the bacteria (Figure 6A). GelMA + CHX 0.2% produced a highly significant inhibition of bacterial growth rate compared to GelMA, GelMA/GelMAQ 50/50%, and GelMA/GelMAQ 25/75%. Furthermore, GelMA/GelMAQ 25/75% produced a slight (10%) but significant inhibition of bacterial growth rate compared to GelMA. The results also show a reduction of 33.72% in the number of UFC/mL at 24 h of the bacteria treated with the Gel that contain more GTMAC (GelMA/GelMAQ 25/75%) compared to the control, but it was not significant (Figure 6B). *P. gingivalis* Live/Dead ratio after 24 h with the different GelMA gels were analyzed to test their effect on bacterial survival. Figure 6C showed a significant decrease in the Live/Dead ratio on GelMA + CHX 0.2% compared to GelMA, GelMA/GelMAQ 50/50%, and GelMA/GelMAQ 25/75%, indicating a bactericidal effect of chlorhexidine, in agreement with previous reports [46].

Last, we also tested gingipain activity after the treatments since *P. gingivalis* gingipains are the main responsible for the extracellular proteolytic activity of the bacteria at the site of infection. Gingipains are potent virulence factors that orchestrate diverse functions, indicating their important role in bacterial clearance, the infection process, activating or inactivating a number of the tissue host proteins, and contributing to the pathogenesis of periodontitis [58]. High gingipain activity is associated with bacterial virulence and plays a key role in tissue degradation. Bacteria treated with GelMA/GelMAQ 25/75% showed significantly lower gingipain activity than all the other treatments (Figure 6C).

Taking together, all the results related to the antibacterial properties of the different GelMA gels tested, it is clear that GelMA + CHX 0.2% produced a high inhibition of bacterial growth rate and a bactericidal effect. This observation is in agreement with the reported effects of CHX, showing strong antimicrobial action [46]. On the other hand, GelMA gels modified with GTMAC showed a slight bacteriostatic effect but significantly decreased the virulence of the bacteria, suggesting that GTMAC could be exerting its activity through the inhibition of bacterial enzymes, such as gingipain activity, although the mechanism of action should be further investigated [59]. According to our study, other reports have demonstrated that GTMAC has antibacterial activity [47], using different materials (chitosan, chitin, chitooligosaccharide, and polyvinyl alcohol (PVA)), showing an inhibition against Gram positive and negative bacteria [47,60,61,62]. The synthesis of GelMA with intrinsic antibacterial properties from quaternary groups is a new alternative to existing gels. Thus, these hydrogels were meant to be different from the typical drug delivery systems. In this case, the antibacterial properties originate from functional groups of the polymer instead of drugs or molecules within the polymer. Hence, there is still the possibility of combining antibacterial drugs with the modified GelMA to get an additive or even synergistic effect.

## 3. Conclusions

In conclusion, a new hydrogel was obtained after the modification of GelMA with quaternary groups with added regenerative, anti-inflammatory, and antibacterial properties. This hydrogel system was fabricated by the mix of GelMA/GelMAQ crosslinked by I2959 and UV light, with easy gelation and stable cross-linking. Furthermore, these gels provide the benefit of in vitro low cytotoxicity and good regenerative properties that typical CHX-based periodontal gels do not provide. Overall, the results showed that the GelMA based hydrogels can be a potentially good alternative option to commercial gels used in the treatment of periodontal diseases.

## 4. Materials and Methods

Materials: Gelatin from porcine skin (type A), methacrylic anhydride (MA) (94%), irgacure I2959 (98%), glycidyltrimethylammonium chloride (GTMAC) (>90%) hydrochloric acid (HCl) (>37%), Ascorbic acid, Water, Benzoyl-arginine pnitroanilide, L-cysteine hydrochloride, and Tris-HCl were purchased from Sigma-Aldrich (St. Louis, MO, USA). Sodium hydroxide (NaOH) (98%) and Brain Heart Infusion (BHI) were purchased from Scharlau (Barcelona, Spain). Dulbecco′s Phosphate Buffered Saline (PBS) was purchased from Capricorn Scientific GmbH (Ebsdorfergrund, Germany). Ham′s F12 (3/1), fetal bovine serum embryonic stem cells tested (FBS), penicillin/streptomycin, HEPES, and Dulbecco′s modified Eagle′s medium (DMEM) low glucose (Biowest, Nuaille, France) were purchased from Biowest (Nuaille, France). LPS from *P. gingivalis* were purchased from InvivoGen (San Diego, CA, USA). Cytotoxicity Detection kit, SYBR green detection, and Lightcycler 480 SYBR Green I Master were purchased from Roche Diagnostics (Mannheim, Germany). Clorhexidine (CHX) was purchased from Abcam (Cambridge, UK). Presto Blue reagent was purchased from Life Technologies (Carlsbad, CA). RNAzol^®^ RT was purchased from Molecular Research Center (Cincinnati, OH, USA). High-Capacity RNA-to-cDNA kit was purchased from Applied Biosystems (Foster City, CA, USA). Immortalized Human Gingival Fibroblasts-hTERT (iHGF) was purchased from Applied Biological Materials Inc. (Richmond, BC, Canada). *P. gingivalis* 33277TM was purchased from (Manassas, VA). Hemin, vitamin K, and Oxoid AnaerogenTM sachet were purchased from Thermo Fisher Scientific (Waltham, MA, USA). LIVE/DEAD BacLight bacterial viability kit was purchased from Invitrogen (Thermo Fisher Scientific, Waltham, MA, USA).

GelMA synthesis (stage 1): In a typical experiment 1.0 g of gelatin (0.286 mmol lysine-functionalities) was dissolved in 60 mL PBS (1.66 % *w*/*w*) at 50 °C separately, after different amounts of MA were added as it is shown in Table 2. The reaction was carried out at 50 °C for 3 h. Then, GelMA was dialyzed for 5 days at 37 °C, lyophilized, and characterized by 1H NMR using deuterium oxide (D2O) as solvent.

GelMA modification (stage 2)*:* In a typical procedure, 0.5 g of GelMA with low DS was dissolved in 8.33 mL distilled water (6% *w*/*w*) at room temperature; then, it was heated at 50 °C, and the solution was alkalinized with [NaOH] = 2 M to Ph = 10. Next, an 8-fold molar excess of GTMAC over free amino groups in GelMA was added. The reaction was carried out at 50 °C for 5 h. Then, the GelMA-quaternary (GelMAQ) was dialyzed for 4 days at 37 °C, lyophilized, and characterized by 1H NMR using D2O as solvent.

Characterization: 1H NMR spectra of polymers were recorded at room temperature in deuterium dioxide (D2O) with a Bruker Advance spectrometer (Billerica, MA, USA) operating at 300 MHz. The proton spectra were used to determine the degree of substitution of primary amine groups by methacryloyl groups of GelMA synthesized with different gelatin/MA molar ratios, and GelMA modification with quaternary ammonium groups from the GTMAC (GelMAQ). The *DS* was calculated from Equation (1), where *A* is the integration area.

(1) $$DS\left(\%\right)=\left[1-\frac{A\left(Lysine\;Methylene\;Groups\;from\;GelMA\right)}{A\left(Lysine\;Methylene\;Groups\;from\;Gelatin\right)}\right]\times 100$$

GelMA hydrogel synthesis: The GelMA hydrogels were synthesized by UV irradiation of the corresponding GelMA in PBS (10% *w*/*w*) in the presence of I2959 (5% *w*/*w* respect to GelMA) as a crosslinker for 2 min. The UV lamp used for the hydrogel synthesis was a UVM-57 Handhel UV Lamp of 6 Watt and wavelength of 302 nm (Upland, CA, USA). The syntheses were carried out under irradiation at a constant distance of 2 cm from the lamp.

The hydrogels were characterized by the swelling *Sw* (%) in PBS (or distilled water) at 37 °C for 24 h in a Nahita Drying Oven (London, UK) and calculated with the Equation (2), where *wh* and *wd* are the weight hydrated and dry.

(2) $$Sw\left(\%\right)=\left(\frac{Wh-Wd}{Wd}\right)\times 100$$

GelMAQ hydrogel synthesis: The GelMAQ hydrogels were synthesized by UV irradiation of the mix of GelMA/GelMAQ 50/50% (*w*/*w*) and GelMA/GelMAQ 25/75% (*w*/*w*) in PBS (10% *w*/*w*) in the presence of I2959 (5% *w*/*w* respect to blend) as crosslinker during 30 and 100 min, respectively. The UV lamp used for the hydrogel synthesis was a UVM-57 Handhel UV Lamp of 6 Watt and wavelength of 302 nm (Upland, CA, USA). The syntheses were carried out under irradiation at a constant distance of 2 cm from the lamp.

Immortalized Human Gingival Fibroblasts cell culture: Immortalized Human Gingival Fibroblasts-hTERT (iHGF) (Applied Biological Materials Inc.) were grown at 37 °C in an atmosphere of 5% CO_2_ using a fibroblast medium, as previously described [46]. iHGF were seeded in 48-well plates at a density of 20,000 cells/well. At confluence, these cells were used for the wound closure assay, cytotoxicity, metabolic activity, and gene expression analysis, using the same methodology as in a previous study [46].

Wound closure assay: The wound was performed as previously described [46]. Afterwards, LPS from *P. gingivalis* (InvivoGen) was added at 1 µg/mL to induce inflammation, and cells were treated with different GelMA compositions at a concentration of 5% (*v*/*v*) in fibroblast medium supplemented with 50 µg/mL ascorbic acid (Sigma-Aldrich, St. Louis, MO, USA) for 48 h. Cells with GelMA treatment served as control, while GelMA with CHX (Abcam) at 0.2% served to compare as a gold standard antibacterial agent. Images of the same areas were taken using a bright-field inverted microscope (Nikon Eclipse TS100) before treatment and 24 and 48 h after treatment, The images were analyzed with the ImageJ software v1.51k (NIH, Bethesda, MD, USA). The wound closure area (%) was defined as: (scratch area at time 0-scratch area at 24 or 48 h)/(scratch area at time 0) × 100. Three independent experiments were performed, with two replicates at each condition (n = 6).

Cell cytotoxicity: Cell culture media was collected after 48 h of the different treatments. Lactate dehydrogenase (LDH) activity was measured with a Cytotoxicity Detection kit (Roche Diagnostics) following the manufacturer′s instructions The results were presented relative to the LDH activity of the media of cells seeded treated with GelMA (control negative, 0% of cell death) and on cells grown on TCP treated with 1% Triton X-100 (control positive, 100% of death), using the following equation: Cytotoxicity (%) = [(expected value-low control)/(high control–low control)] × 100. Three independent experiments were performed, with two replicates at each condition (n = 6).

Metabolic activity: Total metabolic activity was evaluated after 48 h of treatment for iHGF. Presto Blue reagent (Life Technologies) was used at 1 h of reagent incubation time following manufacturer′s protocol. Cells treated with GelMA were set as 100%. Three independent experiments were performed, with two replicates at each condition (n = 6).

Gene Expression by Real-Time RT-PCR: RNA was isolated using RNAzol^®^ RT (Molecular Research Center) from the cell culture monolayer after 48 h of treatment. RNA concentration was quantified with a Nanodrop spectrophotometer (NanoDrop Technologies, Wilmington, DE, USA) and normalized for reverse transcription to cDNA using a High-Capacity RNA-to-cDNA kit (Applied Biosystems).

Real-time PCR was performed for two reference genes, glyceraldehyde-3-phosphate dehydrogenase (GAPDH) and beta-actin (ACTBL2), and several target genes (Table 3).

Real-time RT-PCR was performed in the Lightcycler 480^®^ system using SYBR green detection (Roche Diagnostics) as previously described [46]. All samples were normalized by the mean of the expression levels of reference genes, and changes were related to the GelMA treated group that was set to 100%. Three independent experiments were performed, with two replicates at each condition (n = 6).

Culture and proliferation assay of *P. gingivalis*: *P. gingivalis* 33277TM (ATCC) was grown from frozen stocks on the complete medium of BHI (Scharlab), under anaerobic conditions (10% H_2_, 10% CO_2_ and 80% N_2_) achieved with an Oxoid AnaerogenTM sachet (Thermo Fisher Scientific) at 37 °C for 24–72 h, as previously described [46].

For evaluating the different treatments, 1 mL bacterial suspensions (≈ 3 × 10^8^ bacteria mL) were incubated with the different hydrogels at a 5% (*v*/*v*) concentration in a BHI medium under anaerobic conditions at 37 °C for 24 h, as previously described [46]. Bacterial suspension without treatment served as a control, and GelMA with CHX at 0.2% served as a positive control for bacterial growth inhibition. The optical density (OD) was measured at 600 nm at 0 h and 24 h to determine bacterial proliferation (PowerWave Ht, Biotek instruments, Winooski, VT, USA). Bacterial growth rate (µ) was calculated during the exponential growth phase following the equation ln OD_t24_ − ln ODt_0_ = µ·(t24 − t0). Live/Dead ratio of bacteria was determined after 24 h using the LIVE/DEAD BacLight bacterial viability kit (Invitrogen), following the manufacturer′s instructions. Moreover, bacterial suspensions after 24 h of incubation with the different treatments were serially diluted with PBS, and 100 μL were plated on BHI agar plates; two plates of each dilution were seeded, and two independent experiments were run (n = 6). The plates were incubated for 7–10 days under strictly anaerobic conditions. The number of colonies forming units (CFU) were recorded. Three different experiments were carried out, with two replicates at each condition in each experiment (n = 6).

Gingipain activity of *P. gingivalis*: The proteolytic activity Arg-gingipains of the bacterial suspension with the different treatments, after 24 h of incubation, was determined as previously described [46]. Three independent experiments were performed, with two replicates at each condition (n = 6).

## Figures and Tables

**Table 1:** Degree substitution and swelling for GelMA synthesized with different feed molar ratio.

Molar Ratio Gelatin/MA ^(a)^	DS (%) ^(b)^	Sw (%) ^(c)^
1/0.25	6	1145.0
1/0.5	16	1239.4
1/1	24	1603.5
1/23.5	76	1586.7

^(a)^ Molar ratio calculated from the moiety lysin groups in gelatin (0.286 mmol/1 g) and molecular weight for MA. ^(b)^ Degree of substitution (DS) in gelatin by methacryloyl groups from the MA calculated with Equation (1) using the methylene peaks at δ = 2.9 ppm in the 1H NMR spectra ^(c)^ Swelling (Sw) calculated with Equation (2).

**Table 2:** Molar ratio used for the GelMA synthesis.

Molar Ratio Gelatin/MA	Gelatin (g)/MA (µL)	Concentration Gelatin (mM)/MA (mM)
1/0.25	1/10.7	4.77/1.18
1/0.5	1/21.3	4.77/2.38
1/1	1/42.6	4.77/4.77
1/23.5	1/1000	4.77/111.88

**Table 3:** Genes and primers used in gene expression analysis. Sequence of sense (S) and antisense (A) primers used in the real-time RT-PCR of reference and target genes. Base pairs (bp) [42].

Related Function	Gene	Primer Sequence (5′–3′)	Product Size (bp)
ECM component	Collagen I α1 (COL1A1)	S: CCTGACGCACGGCCAAGAGG A: GGCAGGGCTCGGGTTTCCAC	122
	Decorin (DCN)	S: ATCTCAGCTTTGAGGGCTCC A: GCCTCTCTGTTGAAACGGTC	146
Wound Healing/Fibrogenic	Transforming growth factor-β1 (TGF-B1)	S: TGTCACCGGAGTTGTGCGGC A: GGCCGGTAGTGAACCCGTTG	131
Pro-inflammatory cytokine	Interleukin-6 (IL-6)	S: AGGAGACTTGCCTGGTGAAA A: GCATTTGTGGTTGGGTCAG	196
Reference gene	Glyceraldehyde-3-phosphate dehydrogenase (GAPDH)	S: TGC ACC ACC AAC TGC TTA GC A: AAG GGA CTT CCT GTA ACA A	87
	Beta-Actin (ACTBL2)	S: CTG GAA CGG TGA AGG TGA CA A: AAG GGA CTT CCT GTA ACA A	140

## Data Availability

The data presented in this study are available on request from the corresponding author.
